# Overexpressed tryptophanyl-tRNA synthetase, an angiostatic protein, enhances oral cancer cell invasiveness

**DOI:** 10.18632/oncotarget.4273

**Published:** 2015-06-17

**Authors:** Chien-Wei Lee, Kai-Ping Chang, Yan-Yu Chen, Ying Liang, Chuen Hsueh, Jau-Song Yu, Yu-Sun Chang, Chia-Jung Yu

**Affiliations:** ^1^ Graduate Institute of Biomedical Sciences, College of Medicine, Chang Gung University, Tao-Yuan, Taiwan; ^2^ Department of Cell and Molecular Biology, College of Medicine, Chang Gung University, Tao-Yuan, Taiwan; ^3^ Department of Otolaryngology-Head and Neck Surgery, Chang Gung Memorial Hospital, Lin-Kou, Tao-Yuan, Taiwan; ^4^ Department of Pathology, Chang Gung Memorial Hospital, Lin-Kou, Tao-Yuan, Taiwan; ^5^ Molecular Medicine Research Center, Chang Gung University, Tao-Yuan, Taiwan; ^6^ Pathology Core, Chang Gung University, Tao-Yuan, Taiwan

**Keywords:** tryptophanyl-tRNA synthetase, oral squamous cell carcinoma, migration, invasion

## Abstract

Oral squamous cell carcinoma (OSCC) is one of the most common neoplasms worldwide. Previously, we identified the angiostatic agent tryptophanyl-tRNA synthetase (TrpRS) as a dysregulated protein in OSCC based on a proteomics approach. Herein, we show that TrpRS is overexpressed in OSCC tissues (139/146, 95.2%) compared with adjacent normal tissues and that TrpRS expression positively correlates with tumor stage, overall TNM stage, perineural invasion and tumor depth. Importantly, the TrpRS levels were significantly higher in tumor cells from metastatic lymph nodes than in corresponding primary tumor cells. TrpRS knockdown or treatment with conditioned media obtained from TrpRS-knockdown cells significantly reduced oral cancer cell viability and invasiveness. TrpRS overexpression promoted cell migration and invasion. In addition, the extracellular addition of TrpRS rescued the invasion ability of TrpRS-knockdown cells. Subcellular fractionation and immunofluorescence staining further revealed that TrpRS was distributed on the cell surface, suggesting that secreted TrpRS promotes OSCC progression via an extrinsic pathway. Collectively, our results demonstrated the clinical significance and a novel role of TrpRS in OSCC.

## INTRODUCTION

Oral cancer is one of the most common neoplasms of the head and neck, accounting for 263, 900 newly diagnosed cases and 128, 000 deaths worldwide in 2008 [1]. In Taiwan, the most prevalent area of oral squamous cell carcinoma (OSCC) occurrence worldwide, the incidence rate (increased by 5.82- and 2.35-fold in males and females, respectively) and mortality rate (increased by 2.26-fold) have significantly increased in recent decades [2]. Despite major advances in therapy, the 5-year survival rate of OSCC patients remains less than 50%. Notably, patient outcome is highly associated with tumor stage and metastasis [3–5]. The metastatic processes of OSCC are dependent on the vasculature, nerves or lymphatic vessels adjacent to regional or distal metastases [6, 7], and the extent of OSCC metastasis reflects the efficiency of therapy [8]. Therefore, it is worthwhile to search for tumor markers and to understand their roles in OSCC progression to stratify patients according to their risk of metastasis.

Aminoacyl-tRNA synthetases (ARSs) are essential enzymes responsible for catalyzing the ligation of amino acids to their cognate tRNAs, thereby linking the genetic code to specific amino acids [9]. In addition to serving this canonical function, higher eukaryotic ARSs have been implicated in a variety of non-canonical functions, including transcription, translation, RNA splicing, RNA trafficking, cytokine activity related to inflammation, apoptosis and angiogenesis [10–15]. Several ARSs are associated with human cancers. For example, human lysyl-tRNA synthetase induces cancer cell migration [16], and human glycyl-tRNA synthetase acts against ERK-activated tumor formation [17]. Three ARSs (threonyl-tRNA synthetase, tyrosyl-tRNA synthetase, and aminoacyl-tRNA synthetase-interacting multifunctional protein 1) are documented to function in pro-angiogenic responses, and three ARSs (seryl-tRNA synthetase, tryptophanyl-tRNA synthetase, and glutamyl-prolyl-tRNA synthetase) perform angiostatic functions [18].

Tryptophanyl-tRNA synthetase (TrpRS) is a 53-kDa protein that consists of 471 amino acids, and a mini-TrpRS (residues 48–471) isoform is produced by alternative splicing [19, 20]. Both full-length TrpRS and mini-TrpRS can be mobilized for exocytosis from endothelial cells, and the secreted TrpRS is cleaved by extracellular proteases to produce two additional N-terminally truncated fragments: T1-TrpRS (residues 71–471) and T2-TrpRS (residues 94–471) [21–23]. Full-length, mini- and T1-TrpRS possess aminoacylation activity, but T2-TrpRS lacks this activity. The overexpression of TrpRS has been reported to occur during the development of the salivary gland in *Drosophila* [24], to be associated with delayed-type skin hypersensitivity reactions in guinea pigs [25], to act as a potent antagonist of ocular angiogenesis in a neonatal mouse model [26], and to perform an angiostatic function in human endothelial cells [22]. These studies suggest the multiple functions of TrpRS in various physiological and pathological activities.

Previously, we used laser capture microdissection combined with quantitative proteomic analysis to identify TrpRS as an up-regulated protein in OSCC tissues compared with adjacent normal tissues [27]. However, the clinical and biological significance of TrpRS in OSCC remains unknown. In the present study, we verified the overexpression of TrpRS in OSCC tissues and analyzed the association of the TrpRS expression levels with the clinicopathological characteristics of OSCC patients. We applied gene knockdown, overexpression and extracellular treatments of TrpRS to characterize the phenotypic changes in OSCC cells. We also demonstrated that extracellular TrpRS can bind to the cell surface of OSCC cells. Our study demonstrates the clinical significance of TrpRS in OSCC and provides new insights into TrpRS-mediated OSCC progression.

## RESULTS

### TrpRS is overexpressed and positively correlates with cancer invasiveness in OSCC

**Figure 1:**
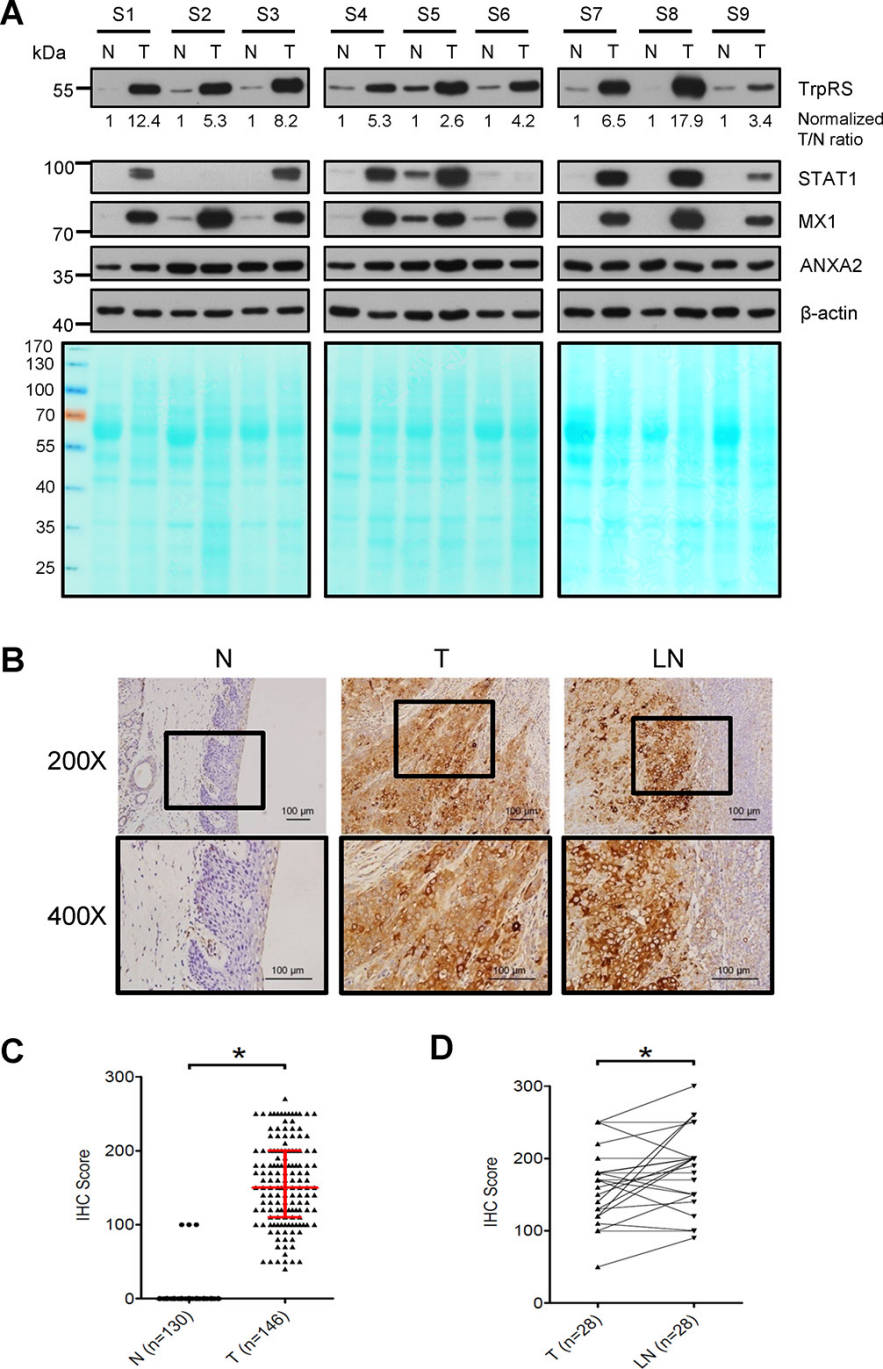
TrpRS is overexpressed in OSCC tissues **A.** Total cell lysates (30 μg per lane) prepared from OSCC tumor tissue (T) and adjacent normal tissue (N) were subjected to SDS-PAGE, transferred to PVDF membrane and then stained with Fast Green FCF dye (lower panel). The proteins were detected by probing with anti-TrpRS, anti-STAT1, anti-MX1, anti-ANXA2 and anti-β-actin antibodies as indicated. β-actin was used as the loading control. The TrpRS signal detected via Western blot was acquired, quantified and normalized to the corresponding β-actin signal. The value represents the normalized fold-change in the tumor tissue relative to the corresponding normal adjacent tissue. **B.** IHC analysis of TrpRS expression in the tumor tissue (T), adjacent normal tissue (N), and the metastatic lymph node tissue (LN) from one representative case. The brown signal indicates the cytosolic distribution of TrpRS in the tumor tissue. **C.** Statistical analysis of the IHC staining scores for TrpRS from 146 paired tumor (T) and adjacent normal tissues (N, 16 normal tissues were missing from the surgically resected OSCC tissue sections). The red line indicates the median with the interquartile range of the IHC staining score. **D.** Statistical analysis of the IHC staining scores for TrpRS from 28 paired tumor tissue (T) and metastatic lymph node (LN) sections. *, a *p* value of less than 0.05 indicates significance based on the paired *t* test.

To verify TrpRS expression in OSCC tissues, we detected the protein levels of TrpRS in paired human OSCC tissues via Western blot and immunohistochemical (IHC) staining. First, a Fast Green FCF dye-stained PVDF membrane image acquired before probing with antibodies was used to visualize the total proteins loaded for Western blot (Figure 1A, lower panel). The β-actin signal was used as the loading control and was applied to obtain a “normalized T/N ratio” to represent the fold-changes of protein expression in the tumor tissue relative to the corresponding adjacent normal tissue. As shown in Figure 1A, the full-length TrpRS was significantly up-regulated (ranged from 2.6 to 17.9) in all of the OSCC tumors (9/9) compared with the corresponding adjacent normal tissues. We also detected three additional proteins, including two up-regulated proteins (STAT1 and MX1) and one unchanged protein (ANXA2), in these paired OSCC tissues based on our previously obtained proteomic dataset [27]. As expected, the expression levels of STAT1 and MX1 were up-regulated in OSCC tumors (7/9 and 9/9 for STAT1 and MX1, respectively), whereas the levels of ANXA2 were similar between the tumor tissues and the adjacent normal tissues. Consistently, IHC analysis demonstrated strong (score > 150) to moderate (score ranged from 50 to 150) TrpRS staining in the cytoplasm of tumor cells but extremely low TrpRS staining in the cells of the adjacent tissue (Figure 1B). The TrpRS levels were dramatically increased in tumor cells, as moderate to strong TrpRS staining was observed in 95.2% (139/146) of the tumors but only 2.3% (3/130) of the adjacent normal tissues (Figure 1C). Furthermore, all 28 lymph node metastatic tissue samples displayed moderate to strong TrpRS staining, and this signal was significantly higher than that detected in the matched primary tumor tissue (*p* < 0.05, Figure 1D). Collectively, these results demonstrated that TrpRS is highly overexpressed in OSCC tissues and that the TrpRS expression level might be associated with cancer invasiveness. Clinicopathological analysis showed that the TrpRS levels in OSCC tumor cells positively correlated with tumor stage, overall TNM stage, perineural invasion and tumor depth (Table 1, *p* < 0.05, Wilcoxon test). There was no significant association between TrpRS level and gender, age or N stage. Based on the IHC staining scores, 144 patients were stratified into two groups (high vs. low expression using a staining score of 150 out of 300 as the cut-off value), and the possible association of TrpRS expression with overall survival (OS) of OSCC patients was evaluated. Survival analysis revealed that the five-year OS rates for patients stratified into the low- and high-TrpRS expression subgroups were 63.0% and 50.7%, respectively (Supplementary Figure S1A). To examine whether the mRNA level of TrpRS is dysregulated in OSCC, we analyzed the gene expression levels of TrpRS in oral cancer tissues compared with normal tissues based on the Oncomine 4.5 database (https://www.oncomine.org). There are seven datasets containing the gene expression level of TrpRS in OSCC cells [28–34]. Importantly, all of these datasets showed that the TrpRS mRNA levels were significantly increased in OSCC tissue compared with normal tissues (Table 2, 2-fold change, *p* value less than 0.05 and ranked in the top 10%). Based on the TrpRS mRNA levels in 31 OSCC patients analyzed by Cromer *et al*. [33], the 5-year OS rates for the patients in the high- and low- TrpRS expression subgroups were 48.0% and 16.7%, respectively (Supplementary Figure S1B). Collectively, these results suggest that elevated TrpRS expression positively correlates with OSCC progression.

**Table 1:** The relationship between TrpRS expression and the clinicopathological characteristics of OSCC patients

Characteristic	Specimens, *n* (%)	TrpRS staining score, mean (SD)^a^	*p* value^b^
Gender			
Female	16 (11.0)	172.5 (57.6)	0.2231
Male	130 (89.0)	153.4 (56.2)	
Age, years			
< 75	71 (48.6)	161.8 (55.2)	0.2543
≥ 75	75 (51.4)	149.5 (57.3)	
Tumor stage			
T1 or T2	77 (52.7)	144.5 (58.8)	0.0152*
T3 or T4	69 (47.3)	167.7 (51.4)	
N stage			
N0	95 (65.1)	151.7 (51.4)	0.2088
N1, N2 or N3	51 (34.9)	162.5 (54.5)	
Overall TNM stage			
I or II	56 (38.4)	136.3 (57.5)	0.0009*
III or IV	90 (61.6)	167.4 (52.6)	
Perineural invasion			
No	102 (70.3)	149.2 (55.7)	0.0486*
Yes	43 (29.7)	169.3 (56.7)	
Tumor depth			
< 8 mm	77 (53.1)	145.7 (57.0)	0.0360*
≥ 8 mm	68 (46.9)	165.9 (54.4)	

a SD, standard deviation.

b *p* < 0.05 indicates significance based on the Wilcoxon test.

**Table 2:** Gene expression levels of TrpRS in OSCC tissues from the Oncomine 4.5 Research Edition Database

Dataset [reference]	Cancer type	Sample size	Fold-change^a^	*p* value
Ginos MA et al. [28]	Buccal mucosa	13		
	Head and neck squamous cell carcinoma	41	5.718	2.36E-18
Peng CH et al. [29]	Normal oral cavity	22		
	Oral cavity squamous cell carcinoma	57	4.137	7.55E-25
Toruner GA et al. [30]	Normal oral cavity	4		
	Oral cavity squamous cell carcinoma	16	3.796	4.38E-05
Pyeon D. et al. [31]	Head and neck normal cell	14		
	Tongue squamous cell carcinoma	15	3.55	2.71E-05
	Oropharyngeal squamous cell carcinoma	6	4.103	2.09E-04
	Tonsillar squamous cell carcinoma	6	3.183	0.003
Ye H. et al. [32]	Normal tongue squamous cell	12		
	Tongue squamous cell carcinoma	26	3.014	9.28E-05
Cromer A. et al. [33]	Normal uvula	4		
	Head and neck squamous cell carcinoma	34	2.642	0.001
Schlingemann J. et al. [34]	Adjacent normal head and neck tissue	4		
	Hypopharyngeal squamous cell carcinoma	4	2.176	0.037

a Fold-changes in mRNA expression in OSCC tissues relative to that in non-cancerous tissues.

### TrpRS knockdown reduces cell viability and oral cancer cell migration and invasion

**Figure 2:**
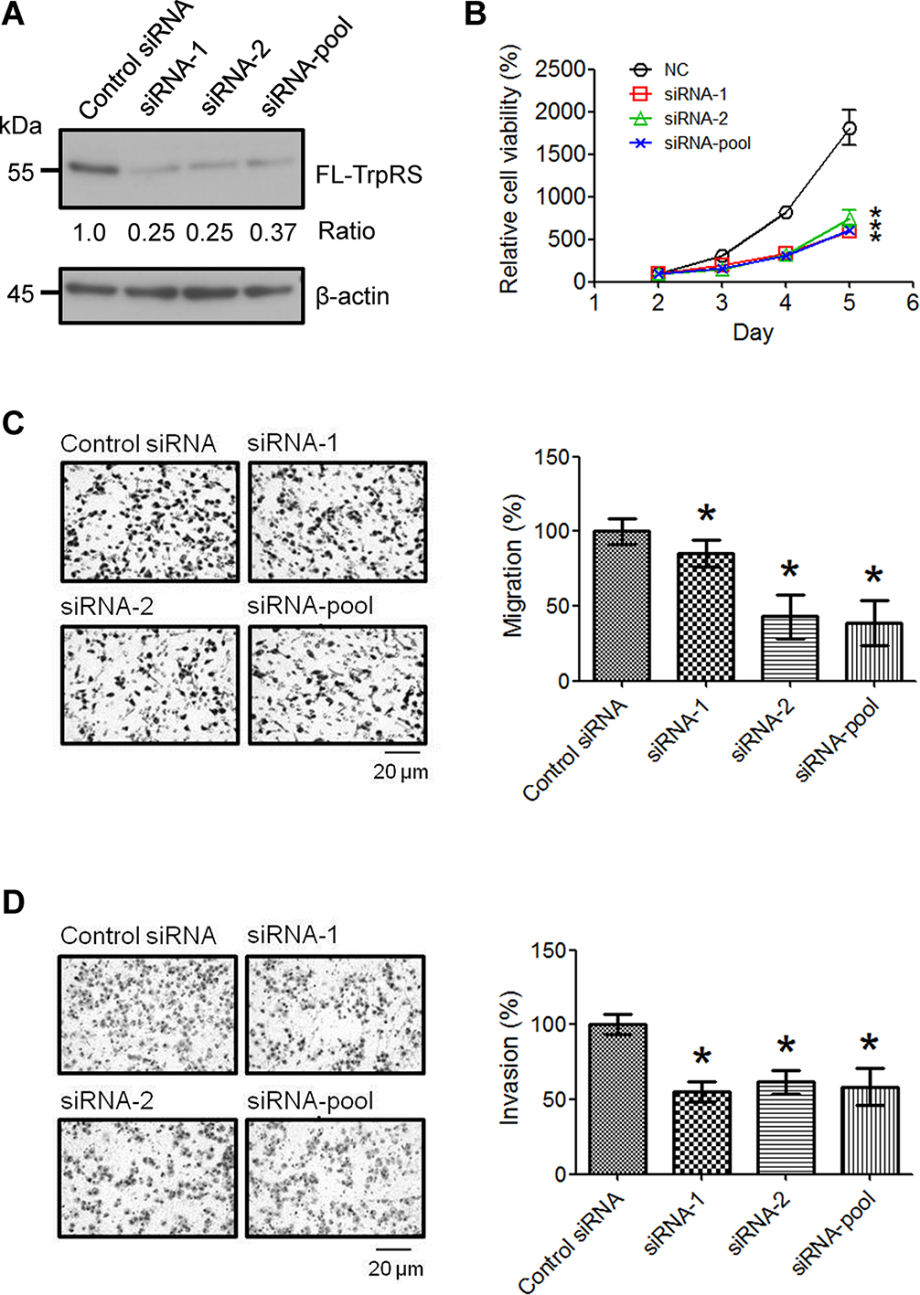
TrpRS knockdown reduces OSCC cell viability, migration and invasion **A.** OEC-M1 cells were transfected with control siRNA or TrpRS-specific siRNA as indicated. At 48 h after transfection, cell lysates were prepared, and the proteins (50 μg per lane) were detected via Western blot. The numbers represent the gene knockdown efficacy of TrpRS-specific siRNA compared with the control siRNA. Simultaneously, cells were subjected to cell counting, migration, and invasion assays as described in the Materials and Methods section. **B.** The quantitative data show the relative percentage of cell viability obtained from three independent cell counting assays. The error bars indicate the standard error of the mean. *, a *p* value of less than 0.05 indicates significance based on two-way ANOVA. Quantitative analysis of the migration **C.** and invasion assays **D.** Images obtained from the migration and invasion assays (left panel). The data are presented as the mean values with standard deviations obtained from three independent experiments (right panel). *, a *p* value of less than 0.05 indicates significance based on the Mann-Whitney *U* test.

To examine the possible role(s) of TrpRS in OSCC tumor progression, we applied siRNA to knock down TrpRS gene expression in oral cancer cells using two distinct siRNA sequences (siRNA-1 and siRNA-2); then, we examined the effects of TrpRS knockdown on cell viability, migration and invasion. Western blot analysis showed that the TrpRS protein levels were significantly reduced to approximately 30% of that in the control cells in TrpRS-knockdown OEC-M1 cells (Figure 2A). Simultaneously, we found that the cell number was significantly reduced in TrpRS-knockdown cells (Figure 2B). The transwell migration assay (6-h incubation) showed that the migration ability of TrpRS-knockdown cells was significantly decreased (85.3%, 43.1% and 38.5% in TrpRS siRNA-1-, siRNA-2- and pooled siRNA-transfected cells, respectively) compared with control cells (Figure 2C). The transwell invasion assay (20-h incubation) also demonstrated that TrpRS knockdown inhibited cell invasion (Figure 2D). A similar result was observed using OC3 cells, as cell viability, migration and invasion abilities were reduced in TrpRS-knockdown OC3 cells (Supplementary Figure S2). There were no changes in cell viability in the OEC-M1 or OC3 cells throughout the migration assay (Supplementary Figure S3). Collectively, these results indicated that TrpRS is involved in OSCC invasiveness.

### TrpRS overexpression promotes cell migration and invasion

**Figure 3:**
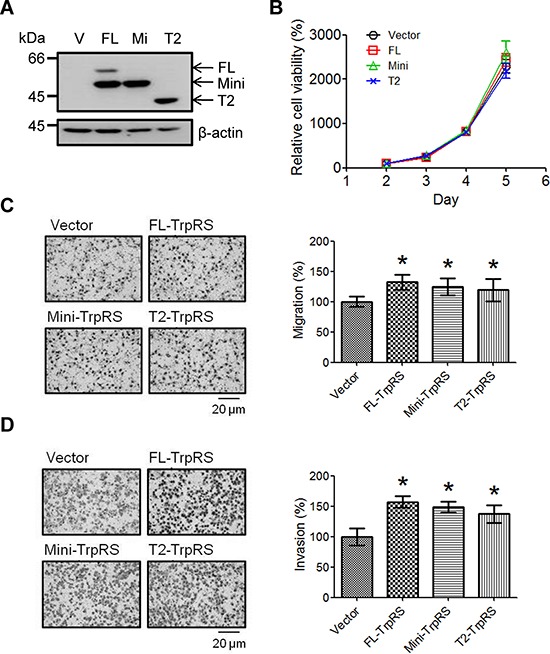
TrpRS overexpression promotes cell migration and invasion **A.** OEC-M1 cells were transfected with the pcDNA 3.1/Myc-His empty vector (V) or a pcDNA 3.1/Myc-His plasmid carrying one of three isoforms of TrpRS (FL: full-length TrpRS; Mi: mini-TrpRS; or T2: T2-TrpRS) as indicated. At 48 h after transfection, cell lysates were prepared, and the proteins were detected via Western blot using an anti-myc antibody. β-actin was used as the loading control. Simultaneously, transfected cells were subjected to cell counting, migration and invasion assays as described in the Materials and Methods section. **B.** Quantitative data show the relative percentage of cell viability obtained from three independent cell counting assays. The error bars indicate the standard error of the mean. Quantitative analysis of the migration **C.** and invasion assays **D.** Photographs obtained from the migration and invasion assays (left panel). The data are presented as values with standard deviations obtained from three independent experiments (right panel). *, a *p* value of less than 0.05 indicates significance based on the Mann-Whitney *U* test.

To confirm the function of TrpRS in OSCC cells, three plasmids (full-length, mini- or T2-TrpRS) were constructed and expressed in OEC-M1 cells. The exogenously expressed TrpRS isoforms were detected via Western blot as expected, although we also detected a potential mini-TrpRS protein band in the full-length TrpRS-expressing cells (Figure 3A). Figure 3B shows that the overexpression of full-length, mini- or T2-TrpRS did not promote the growth of OEC-M1 cells compared with the cells transfected with an empty vector. As expected, we found that the overexpression of full-length, mini- or T2-TrpRS promoted the migration and invasion abilities of OEC-M1 cells compared with empty vector transfection (Figure 3C and 3D). These results suggest that TrpRS overexpression promotes the migration and invasion in oral cancer cells.

### Secreted TrpRS promotes oral cancer cell invasion

**Figure 4:**
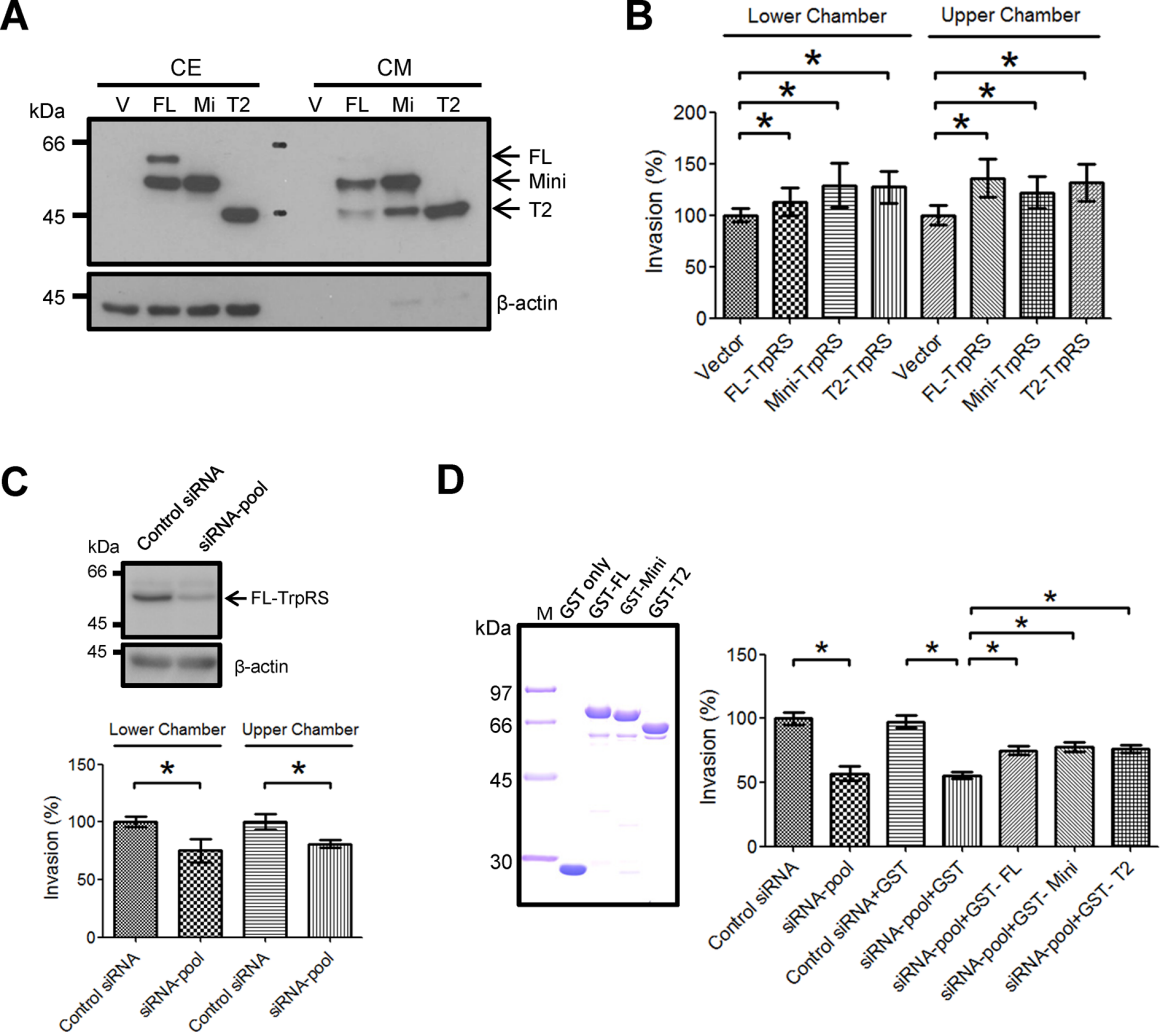
Secreted TrpRS promotes OSCC cell invasion **A.** The detection of secreted TrpRS in CM via Western blot. Cell extracts (CE; 50 μg) and CM (10 μg) obtained from transfected OEC-M1 cells (V: empty vector; FL: full-length TrpRS; Mi: mini-TrpRS; T2: T2-TrpRS) were prepared for Western blot using an anti-myc antibody. β-actin was used as the loading control. **B.** The treatment of recipient cells with CM harvested from TrpRS-expressing cells promoted cell invasion. The recipient OEC-M1 cells were treated with CM harvested from TrpRS-expressing cells throughout the invasion assay. **C.** The treatment of recipient cells with CM harvested from TrpRS-knockdown cells reduced cell invasion. CE from TrpRS-knockdown OEC-M1 cells were prepared for Western blot using an anti-TrpRS antibody. β-actin was used as the loading control. The recipient OEC-M1 cells were treated with CM harvested from TrpRS-knockdown cells throughout the invasion assay. **D.** The extracellular addition of recombinant TrpRS protein rescued the invasion ability of TrpRS-knockdown oral cancer cells. Purified GST-TrpRS fusion proteins (2 μg per lane) were separated via SDS-PAGE and stained with Coomassie Brilliant Blue (left panel). The TrpRS-knockdown OSCC cells were treated with a GST-TrpRS fusion protein (1 μg/ml) throughout the invasion assay. The quantitative analysis of the invasion assays (C–D) is presented as the mean values with standard deviations obtained from three independent experiments. *, a *p* value of less than 0.05 indicates significance based on the Mann-Whitney *U* test.

To understand the role of secreted TrpRS in OSCC cells, we detected the secretion of exogenously expressed TrpRS in the conditioned media (CM) of oral cancer cells and examined the effects of secreted TrpRS on the recipient OSCC cell invasiveness cells. Figure 4A shows the expression and secretion of the three TrpRS isoforms (full-length-, mini- and T2-TrpRS) in cell extracts and CM. T2-TrpRS was detected in CM derived from all three TrpRS isoform-transfected cells, supporting the extracellular proteolysis of the T2 isoform in OSCC cells. To evaluate whether secreted TrpRS acts as a cytokine-like protein to affect oral cell invasiveness, CM were applied to either the lower or upper chamber of transwell devices and an invasion assay was performed. We observed that compared with CM from empty vector-transfected cells, CM harvested from full-length-, mini- or T2-TrpRS-expressing cells increased the invasiveness of recipient cells when the CM was added to the lower or upper chamber (Figure 4B). To confirm this effect, we performed similar experiments using CM harvested from TrpRS-knockdown cells. Figure 4C shows that the treatment of recipient cells with CM harvested from TrpRS-knockdown cells indeed significantly reduced cell invasiveness regardless of the CM application site. Collectively, these results suggest that secreted TrpRS promotes OSCC cell invasion.

### Extracellular treatment of TrpRS promotes cell invasion in oral cancer cells

To determine whether secreted TrpRS is sufficient to promote OSCC cell invasion, we generated recombinant glutathione-S-transferase (GST)-TrpRS full-length-, mini- or T2-TrpRS fusion proteins in *E. coli* and examined their effects on cell invasion. The quality and purity of the purified GST-TrpRS fusion proteins were confirmed via SDS-PAGE followed by Coomassie Brilliant Blue staining (Figure 4D, left panel). The extracellular treatments of each GST-TrpRS fusion protein promoted cell migration, confirming its biological activity in promoting cell invasiveness (Supplementary Figure S4). Figure 4D shows that with or without GST control protein treatment, the invasiveness of TrpRS-knockdown cells was similar (56.8% *vs*. 55.4% of control cells). After the treatment of TrpRS-knockdown cells with the GST-full-length TrpRS, GST-mini-TrpRS or GST-T2-TrpRS fusion protein, cell invasion was significantly rescued to 75.1%, 77.6% and 76.1% of the control levels, respectively (Figure 4D, right panel). This result suggests that the extracellular treatment of TrpRS promotes OSCC cell invasion.

### Detection of TrpRS on the cell surface of OSCC cells

**Figure 5:**
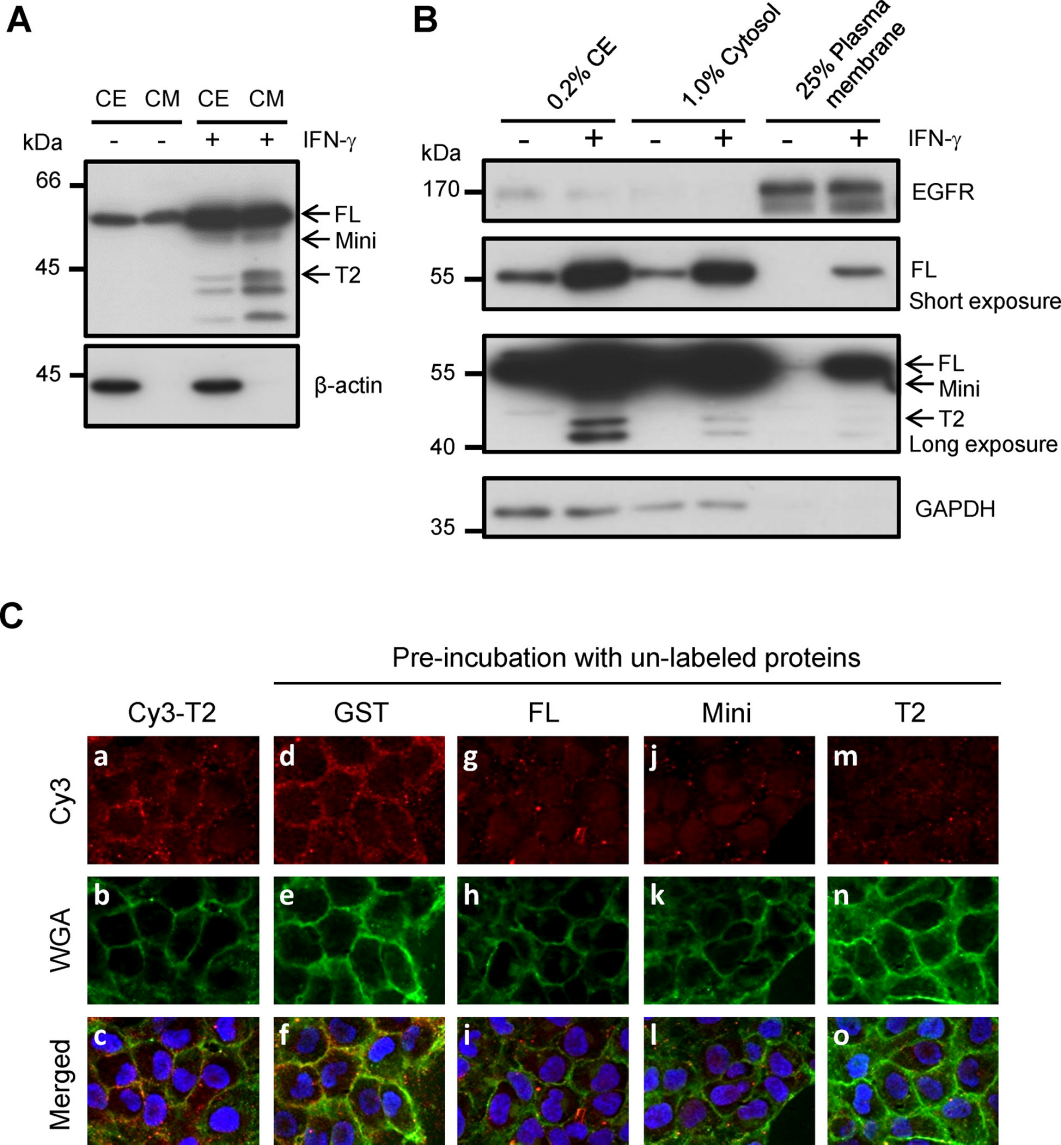
The detection of surface-bound TrpRS on INF-γ-treated OSCC cells **A.** The detection of intracellular and extracellular TrpRS expressions in IFN-γ-treated OSCC cells. OEC-M1 cells were treated with IFN-γ (200 U/ml) for 24 h. Cell extracts (CE) and CM were obtained from control and IFN-γ treated cells, and the proteins were detected via Western blot using an anti-TrpRS antibody. β-actin was used as the loading control. **B.** TrpRS was detected in the plasma membrane (PM) fraction of IFN-γ-treated OEC-M1 cells. OEC-M1 cells were treated with IFN-γ (200 U/ml) for 24 h. The whole-cell extract (CE), cytosolic (Cytosol) and PM fractions were prepared as described in the Materials and Methods section. The proteins were subjected to Western blot using anti-EGFR, anti-TrpRS and anti-GAPDH antibodies as indicated. **C.** Cy3-labeled T2-TrpRS was detected on the cell surface via immunofluorescence staining as described in the Materials and Methods section. Cells pre-incubated without **a–c.** or with 50 μg/ml unlabeled GST **d–f.** full-length GST-TrpRS **g–i.** GST-mini-TrpRS **j–l.** or GST-T2-TrpRS **m–o.** are presented. The Cy3-labeled T2-TrpRS (red), WGA (green) and merged images are presented as indicated. DNA was stained with Hoechst 33258 (blue).

Based on the finding that extracellular TrpRS promotes OSCC cell invasiveness, we hypothesized that secreted TrpRS binds to unidentified receptors or associated proteins on the plasma membrane (PM), thereby triggering downstream signaling to promote cell invasiveness. To test this hypothesis, we examined the binding of secreted TrpRS to the OSCC cell surface using PM protein fractionation and immunofluorescence staining. As shown in Figure 5A, TrpRS expression and secretion were notably increased in interferon-gamma (IFN-γ)-treated oral cancer cells compared with control cells. We detected full-length TrpRS and only a small amount of the mini- and T2-TrpRS proteins in the PM fraction after IFN-γ treatment (Figure 5B). Immunofluorescence staining demonstrated that Cy3-labeled T2-TrpRS was distributed on the cell surface of OSCC cells, where it partially co-localized with the Alexa Fluor^®^ 488 conjugate WGA on the cell surface (Figure 5C, 5a–5c). An inhibition assay further confirmed the specific binding of TrpRS to the cell surface that the cell surface binding signals of Cy3-labeled T2-TrpRS were suppressed by pre-incubation with the unlabeled GST-full-length TrpRS (Figure 5C, 5g–5i), GST-mini-TrpRS (Figure 5C, 5j–5l) and GST-T2-TrpRS fusion proteins (Figure 5C, 5m–5o) but not GST protein alone (Figure 5C, 5d–5f). These results imply that secreted TrpRS promotes cell invasiveness, possibly via its binding to the cell surface of oral cancer cells.

## DISCUSSION

To our knowledge, our study is the first to characterize the clinical and biological significance of TrpRS in OSCC. TrpRS expression can be induced by interferon stimulation in various cell types [19, 35, 36]. Our previous study demonstrated that interferon signaling was significantly altered in OSCC lesions and that the motility of OSCC cells was increased in response to interferon treatment. In the present study, we show that overexpressed TrpRS in OSCC tissue promotes cell migration/invasion, supporting the hypothesis that TrpRS is triggered by interferon and is involved in interferon-enhanced cellular mobility.

TrpRS is dysregulated in different cancers, including ovarian, cervical, colorectal and pancreatic cancers. In ovarian cancer, the TrpRS protein levels were up-regulated in highly malignant clear cell adenocarcinoma compared with mucinous ovarian adenocarcinoma with a low malignant potential [37]. Increased expression of TrpRS was found in cervical carcinoma tissue compared with normal tissue [38]. In contrast, a low expression level of TrpRS correlated with an increased risk for lymph node metastasis and a more advanced tumor stage in colorectal cancer [39]. Recently, Paley *et al*. showed that pronounced down-regulation of full-length TrpRS caused by hypoxia is concomitant with increased metastatic ability [40]. In the present study, we observed that the TrpRS level was up-regulated in OSCC and was associated with cancer invasiveness. These findings suggest that TrpRS exerts a paradoxical effect on tumor invasiveness in different types of cancer. Considering that truncated TrpRS performs an angiostatic function in endothelial cells but that the TrpRS expression level was increased and positively correlated with cancer invasiveness in OSCC, in this study, we hypothesized that TrpRS acts as an angiostatic and oncogenic factor in OSCC. Although the underlying mechanism requires further study, we propose that the alteration of the interferon signaling pathway, which promotes cancer motility in OSCC cells, might account for at least part of this discrepancy.

The non-canonical functions of ARSs have emerged as attractive therapeutic targets for diseases such as inflammation, tumorigenesis, and angiogenesis and other important physiopathological processes [41, 42]. Notably, anti-angiogenic therapy elicits the malignant progression of tumors, leading to increased local invasion and distant metastasis, as reported by Paez-Ribes *et al.* [43]. Ebos *et al*. also reported accelerated metastasis after short-term treatment with a potent inhibitor of tumor angiogenesis, supporting the limitation of anti-angiogenic therapies based on clinical trials [44, 45]. These studies revealed that a potent angiogenesis inhibitor might alter the natural history of tumors by increasing invasion and metastasis. One reason that may account for this phenomenon is that cancer cells might become more aggressive to survive in or escape from an unfavorable environment (e.g., low oxygen and low nutrient levels). We proposed that TrpRS acts as a natural angiostatic agent and an oncogenic factor in OSCC to exert opposing effects on cancer progression, which might result in the limited correlation between TrpRS expression and OSCC patient survival in the present study (Supplementary Figure S1).

It is well characterized that secreted TrpRS promotes angiostatic potential in endothelial cells until the removal of the N-terminal domain of full-length TrpRS. Specifically, the truncated form of TrpRS, T2-TrpRS, binds to VE-cadherin [46] and blocks the VEGF-mediated survival signaling of AKT in endothelial cells, thereby performing an angiostatic function [47]. In the present study, we found that the extracellular treatment of either CM from TrpRS-transfected cells or recombinant TrpRS isoforms enhanced OSCC invasiveness (Figure 4 and Supplementary Figure S4). To date, there is no published study of the specific receptor(s) or molecule(s) that interact with TrpRS on the surface of epithelial cells. Future investigation is warranted to identify the receptor(s) or associated molecule(s) that mediate TrpRS-regulated cell invasiveness. In addition, developing non-invasive methods to determine the levels of TrpRS in bodily fluids (e.g., saliva or serum) would be useful for OSCC diagnosis/prognosis in clinical practice. These methods include enzyme-linked immunosorbent assays and mass spectrometric multiple reaction monitoring-based strategies. The mass spectrometric strategies could be developed for this purpose to identify or quantify isoform-specific peptides in a multi-targeted manner [48]. However, the distinct functions of the TrpRS isoforms in OSCC tumorigenesis should be elucidated to understand the clinical application of TrpRS isoforms to OSCC diagnosis or prognosis.

In conclusion, we present for the first time the clinical significance and biological function of TrpRS in OSCC. Both the mRNA and protein levels of TrpRS isoforms were upregulated in OSCC, and the TrpRS levels positively correlated with OSCC invasiveness. In addition to its well-characterized angiostatic function in endothelial cells, TrpRS is involved in OSCC progression. Our results also suggest that secreted TrpRS promotes cell invasiveness by extrinsically binding to unidentified receptor(s) or associated molecule(s) on the cell surface. Collectively, our findings provide new insights into TrpRS-mediated OSCC tumorigenesis.

## MATERIALS AND METHODS

### Cell culture

The human oral epidermal carcinoma cell line OEC-M1 was derived from the gingiva of a Chinese patient and was maintained in RPMI 1640 medium (Invitrogen, Carlsbad, CA, USA) containing 10% fetal bovine serum (FBS), 25 mM HEPES and antibiotics as previously described [49]. The cells were cultured at 37°C in a humidified atmosphere of 95% air/5% CO_2_.

### Antibodies

The commercially available primary antibodies used in this study included the following: polyclonal rabbit anti-TrpRS (Abnova, Taipei, Taiwan); polyclonal rabbit anti-MX1 (Abcam, Cambridge, UK); monoclonal mouse anti-STAT1 (BD Biosciences); monoclonal mouse anti-ANXA2 (R&D, Eugene, OR, USA); and monoclonal mouse anti-β-actin and anti-myc (Millipore, Billerica, MA, USA). The secondary antibodies used for Western blot included HRP-conjugated goat anti-rabbit and anti-mouse IgG antibodies were purchased from GE Healthcare.

### Patient populations and clinical specimens

Written informed consent was acquired from all of the patients enrolled in this study before sample collection, and this study was approved by the Institutional Review Board of Chang Gung Memorial Hospital (CGMH), Tao-Yuan, Taiwan. For Western blot, nine surgically resected OSCC and adjacent normal tissues (8 males and 1 female; age range, 37–65 years) were obtained from patients who underwent surgery at CGMH. Tumor specimens for IHC analysis were obtained from 157 surgically resected OSCC tumors together with the adjacent normal epithelial tissue in a consecutive cohort of OSCC patients diagnosed at CGMH (Tao-Yuan, Taiwan) from 2002 to 2007. The patients in this study underwent standard preoperative assessment according to the institutional guidelines, including a detailed medical history, complete physical examination, computed tomography or magnetic resonance imaging scans of the head and neck, chest radiographs, bone scans, and abdominal ultrasounds. Primary tumors were excised with adequate margins under intraoperative frozen section control. After surgical treatment, the pathological and nodal stages of all tumors were determined according to the AJCC Cancer Staging Manual (2010).

### IHC staining and scoring

IHC was performed as described previously [50]. Consecutive sections (5-μm thickness) of formalin-fixed paraffin-embedded specimens from OSCC patients were subjected to IHC analysis using a rabbit anti-TrpRS polyclonal antibody (Abnova) using an automated IHC device according to the manufacturer's instructions (Bond™, Vision Biosystems, Mount Waverley, VIC, Australia). The expression level of TrpRS was scored using a combined method accounting for both the staining intensity and the percentage of stained cells. The resulting combined score was calculated as the sum of the percentage of stained cells multiplied by the intensity score [51, 52]. According to the sum of the IHC score (0–300), the level of TrpRS was categorized into groups of negative (0), weak (<50), moderate (50–150) and strong immunostaining (>150). The specimens were independently evaluated by pathologists without prior knowledge of the clinical data.

### Gene knockdown of TrpRS using siRNA

siRNA duplexes targeting human TrpRS were synthesized and developed using the highly effective Stealth RNAi™ siRNA technology from Invitrogen. The siRNA sequences used are as follows: siRNA-1, UAUCCGUCCUGUCUCGGAAGAUCUG; siRNA-2, UAUGAGUGCCUUCUUGAGCUCACCG. For gene knockdown, OEC-M1 cells were transfected with siRNA using Lipofectamine™ RNAiMAX transfection reagent (Invitrogen) according to the manufacturer's instructions. After 48 h of transfection, cell extracts were collected, and the knockdown efficacy was verified by Western blot.

### Cloning and gene expression of TrpRS

The plasmids encoding full-length TrpRS were constructed via polymerase chain reaction (PCR) using the sense primer 5′-ATGCCCAACAGTGAGCCCGCAT-3′ and the antisense primer 5′-CTGAAAGTCGAAGGACA GCTTC-3′. The full-length TrpRS gene fragment was inserted into the pGEM-T easy vector (Promega Corporation, Madison, WI, USA). The plasmids encoding mini- and T2-TrpRS were amplified from the full-length TrpRS via PCR using the sense primer 5′-TCTAGAATGAGCTACAAAGCTGC-3′ and the antisense primer 5′-TCTAGACTGAAAGTCGA AGGAC-3′ for mini-TrpRS or the sense primer 5′-TCTAGAATGAGTGCAAAAGGCATA-3′, and the antisense primer 5′-TCTAGACTGAAAGTCGAAGGAC-3′ for T2-TrpRS. To generate the myc-tagged constructs for expression in OEC-M1 cells, the TrpRS gene fragments were subcloned into the pcDNA3.1/Myc-His plasmid (Invitrogen) and then transfected using Lipofectamine™ 2000 (Invitrogen) according to the manufacturer's protocol. After 48 h of transfection, the cells were collected for further functional assays. To generate the GST fusion constructs for expression in *E. coli*, we subcloned the fragments encoding these TrpRS isoforms into the pGEX4T3 vector (GE Healthcare, Little Chalfont, Buckinghamshire, United Kingdom).

### Expression and purification of recombinant TrpRS protein from *E. coli*

The TrpRS/pGEX4T3 constructs were transformed into BL21 (DE3) cells and cultured in LB medium containing ampicillin (50 μg/ml). The GST-TrpRS fusion proteins were synthesized in mid-log phase *E. coli* via induction using 0.5 mM IPTG for 3 h at 37°C. The soluble GST-TrpRS fusion proteins were purified from the culture media using Glutathione Sepharose™ 4 Fast Flow resins (GE Healthcare, Uppsala, Sweden) according to the manufacturer's instructions.

### The binding of Cy3-labeled TrpRS to the surface of OSCC cells

GST and the GST-TrpRS fusion proteins were labeled with the Cy3 Mono-Reactive Dye Pack (GE Healthcare, Little Chalfont, Buckinghamshire, United Kingdom) according to the manufacturer's instructions. Briefly, 1 mg of protein (2 μg/μl) in 0.1 M sodium carbonate buffer, pH 9.3, was labeled at room temperature for 30 min. The Cy3-labeled proteins were purified using a HiTrap desalting column (Sephadex™ G-25 Superfine, 1.6 × 2.5 cm, GE Healthcare, Uppsala, Sweden) connected to an ÄKTA purifier-10 fast performance liquid chromatograph (GE Healthcare, Uppsala, Sweden). For the protein-binding assay, OEC-M1 cells were seeded in 12-well plates on cover slides and cultured for 48 h to 70% confluence. The cells were pre-incubated in serum-free RPMI medium with or without 50 μg/ml unlabeled GST or various forms of GST-TrpRS (full-length, mini- or T2-TrpRS) at 4°C for 15 min. Subsequently, Cy3-labeled GST or Cy3-labeled GST-TrpRS fusion protein (10 μg/ml) was added to the pre-incubated media at 4°C for additional 15 min. The cells were washed with cold PBS and then fixed with 4% formaldehyde for 15 min, followed by WGA-488 labeling (Alexa Fluor^®^ 488 conjugate, Invitrogen). The cells were washed with PBS and mounted using a mounting solution that contained Hoechst 33258. Images were acquired using a Zeiss ApoTome fluorescence microscope and AxioVision Release 4.8 software (Carl Zeiss, Gottingen, Germany).

### Cell viability analysis

The viability of OSCC cells was determined by cell counting. Briefly, cells transfected with siRNA or plasmids for 24 h were plated on 12-well plates (1 × 10^4^ cells/well) and cultured for the indicated periods. The cells were harvested and re-suspended in PBS, and 10 μl of the cell suspensions were mixed with an equal volume of Trypan blue for cell counting. The number of living cells was determined using a TC20™ Automated Cell Counter (Bio-Rad, Hercules, CA, USA) according to the manufacturer's instructions. Each data point for the cell number represents triplicate determinations from three independent experiments.

### Cell migration and invasion assays

Cells transfected with siRNA or plasmids for 48 h were harvested via trypsinization and suspended in serum-free culture medium. For the migration assay, the cells (300 μl; 7.5 × 10^4^ cells) were added to the upper chamber of 24-well transwell chambers (0.8-μm pore size filter; Corning, Canton, NY, USA), and each lower chamber was filled with 600 μl of serum-free culture medium containing 10 μl/ml fibronectin. After a 6-h incubation at 37°C, the chambers were gently washed twice with PBS and fixed with 100% methanol for 15 min, followed by Giemsa staining. The cells that had traversed the filter to the lower chamber were counted microscopically in 6 different fields per filter. For the invasion assay, the upper chamber of 24-well transwell chambers was coated with Matrigel™ Basement Membrane Matrix (BD Biosciences, San Jose, CA, USA) at 37°C for 1 h. The cells (1 × 10^5^ cells) were suspended in 200 μl of serum-free culture medium and added to the upper chamber, and each lower chamber was filled with 600 μl of serum-free culture medium. After a 20-h incubation at 37°C, the chambers were washed, fixed, stained and counted as described above.

### Preparation of CM from OSCC cells

CM were collected and processed as previously described [50]. Briefly, OEC-M1 cells were grown to 90% confluence in 10-cm culture dishes, washed with serum-free media and then cultured in serum-free media for 24 h. CM were subsequently collected and centrifuged at 1000 g for 10 min to remove the cell pellet and debris. The supernatants were concentrated and desalted via centrifugation in an Amicon Ultracel tube (10 kDa molecular weight cutoff; Millipore, Billerica, MA, USA). The protein concentrations were determined using the Bradford protein assay (Bio-Rad).

### Extraction of PM proteins from OSCC cells

PM proteins were extracted using the Plasma Membrane Protein Extraction Kit (Abcam) according to the manufacturer's protocol. Briefly, OEC-M1 cells (5 × 10^8^) treated or not treated with IFN-γ (200 U/ml; PeproTech, Inc., Rocky Hill, NJ, USA) for 24 h were collected via gentle scraping in PBS. The whole-cell extract (CE) was obtained after a PBS wash, followed by homogenization in 1 ml of homogenization buffer. The cytosolic fraction and the total membrane protein fraction were prepared via sequential centrifugation. The PM proteins were then recovered via extraction from two-phase solution (upper and lower) separation using stepwise centrifugation. Finally, the PM proteins were dissolved in 0.5% Triton X-100 for further analysis.

### Statistical analyses

GraphPad Prism (GraphPad Software, Inc., La Jolla, CA, USA) was used to perform statistical analysis of the data from the functional assays. The nonparametric Mann-Whitney *U* test was employed to analyze the data from the transwell migration and invasion assays. For quantitative analysis of cell viability, two-way ANOVA was used. The clinical significance of TrpRS was assessed using SAS software (SAS Institute, Inc., Cary, NC, USA). The relationship between TrpRS expression and clinicopathologic characteristics was analyzed using the Wilcoxon test. All *p* values less than 0.05 were considered to be statistically significant.

## SUPPLEMENTARY FIGURES


