# Supplementary material for: Overexpressed tryptophanyl-tRNA synthetase, an angiostatic protein, enhances oral cancer cell invasiveness
Source: Oncotarget. 2015 Jun 17;6(26):21979–92. doi: 10.18632/oncotarget.4273 (PMC4673140; doi:10.18632/oncotarget.4273)
Supplement: Supplementary file 1 [file oncotarget-06-21979-s001.pdf]

SUPPLEMENTARY FIGURES

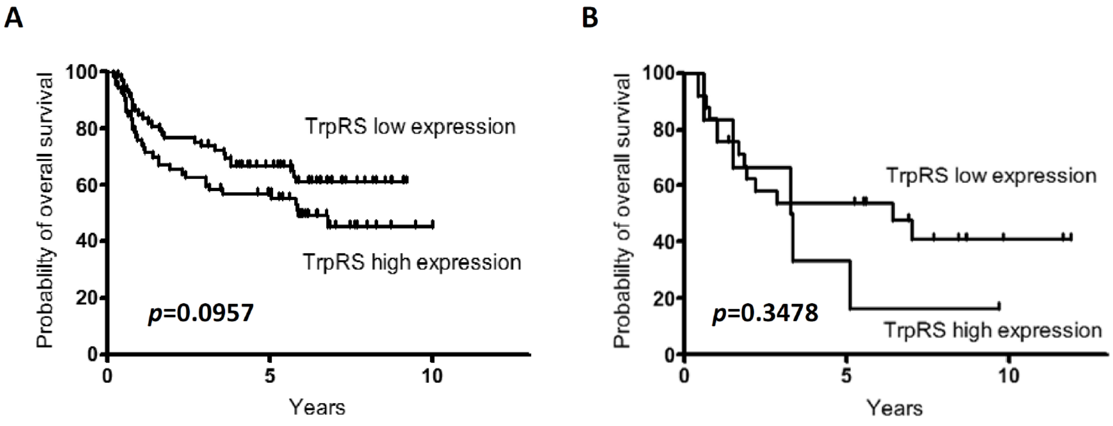

Supplementary Figure S1: TrpRS overexpression correlates with poor patient survival. A. The TrpRS protein expression levels in 144 OSCC patients were determined via IHC analysis and subjected to survival analysis in the present study. The Kaplan-Meier plot shows that the 5-year overall survival rates for patients stratified into low- and high-TrpRS expression subgroups were 63.0% and 50.7%, respectively (*p* = 0.0957, log-rank test). **B.** The TrpRS mRNA expression levels in 31 OSCC patients were determined via microarray analysis [1] and subjected to survival analysis. The Kaplan-Meier plot shows that the 5-year overall survival rates for patients stratified into low- and high-TrpRS expression subgroups were 48.0% and 16.7%, respectively (*p* = 0.3478, log-rank test).

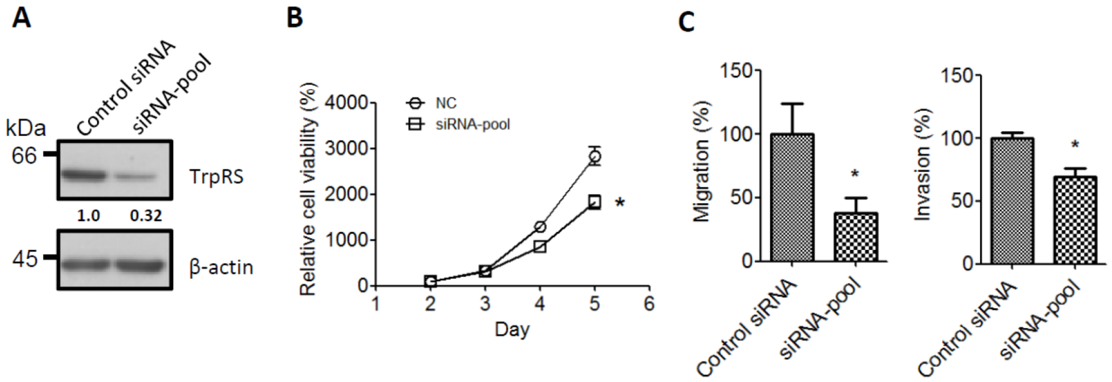

Supplementary Figure S2: TrpRS knockdown reduces OC3 cell viability, migration and invasion. A. OC3 cells were transfected with control siRNA or TrpRS-specific siRNA as indicated. At 48 h after transfection, cell lysates were prepared, and the proteins (50 µg per lane) were detected via Western blot. The numbers represent the gene knockdown efficacy of TrpRS-specific siRNA relative to control siRNA. Simultaneously, cells were subjected to cell counting, migration, and invasion assays as described in the Materials and Methods section. **B.** The quantitative data show the relative percentage of cell viability obtained from three independent cell counting assays. The error bars indicate the standard error of the mean. *, a *p* value of less than 0.05 indicates statistical significance based on the two-way ANOVA. **C.** Quantitative analysis of the migration and invasion assays. The data are presented as the mean values with standard deviations obtained from three independent experiments. *, a *p* value of less than 0.05 indicates statistical significance based on the Mann-Whitney *U* test.

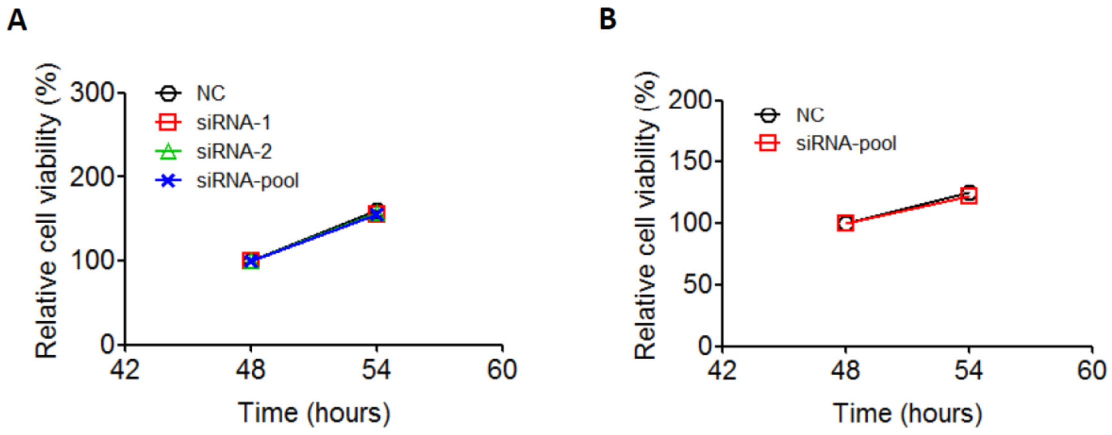

Supplementary Figure S3: Cell viability in TrpRS-knockdown cells throughout the migration and invasion assays. OEC-M1 **A.** or OC–3 **B.** cells were transfected with control siRNA, siRNA-1, siRNA-2 or siRNA-pool as indicated. The quantitative data show the relative percentage of cell viability obtained from three independent cell counting assays. The error bars indicate the standard error of the mean.

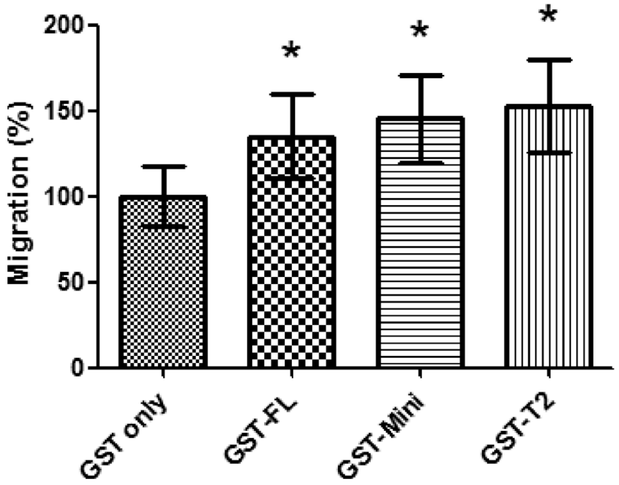

Supplementary Figure S4: *In vitro* treatment with GST-TrpRS fusion proteins promotes the migration of OEC-M1 cells. The expression and purification of GST-TrpRS fusion proteins were described in the Materials and Methods section. Quantitative analysis of the migration assay of mock OEC-M1 cells subjected to *in vitro* treatment with GST-TrpRS fusion protein (1 μg/ml). Cells (7.5×10^4) were applied to the upper chamber of a transwell chamber containing 200 μl of Opti-MEM. The data are presented as the mean values with standard deviations obtained from three independent experiments. *, a p value of less than 0.05 indicates statistical significance based on the Mann-Whitney U test.

REFERENCES

1. Cromer A, Carles A, Millon R, Ganguli G, Chalmel F, Lemaire F, Young J, Dembele D, Thibault C, Muller D, Poch O, Abecassis J, Wasylyk B. Identification of genes associated with tumorigenesis and metastatic potential of hypopharyngeal cancer by microarray analysis. Oncogene. 2004; 23:2484–2498.
